# ^1^Progress, applications, challenges and prospects of protein purification technology

**DOI:** 10.3389/fbioe.2022.1028691

**Published:** 2022-12-06

**Authors:** Miao Du, Zhuru Hou, Ling Liu, Yan Xuan, Xiaocong Chen, Lei Fan, Zhuoxi Li, Benjin Xu

**Affiliations:** ^1^ Department of Medical Laboratory Science, Fenyang College, Shanxi Medical University, Fenyang, China; ^2^ Science and Technology Centre, Fenyang College of Shanxi Medical University, Fenyang, China; ^3^ Key Laboratory of Lvliang for Clinical Molecular Diagnostics, Fenyang, China; ^4^ Department of Basic Medicine, Fenyang College of Shanxi Medical University, Fenyang, China

**Keywords:** protein purification, challenges and prospects, life and health industry, affinity chromatography, ultracentrifugation

## Abstract

Protein is one of the most important biological macromolecules in life, which plays a vital role in cell growth, development, movement, heredity, reproduction and other life activities. High quality isolation and purification is an essential step in the study of the structure and function of target proteins. Therefore, the development of protein purification technologies has great theoretical and practical significance in exploring the laws of life activities and guiding production practice. Up to now, there is no forthcoming method to extract any proteins from a complex system, and the field of protein purification still faces significant opportunities and challenges. Conventional protein purification generally includes three steps: pretreatment, rough fractionation, and fine fractionation. Each of the steps will significantly affect the purity, yield and the activity of target proteins. The present review focuses on the principle and process of protein purification, recent advances, and the applications of these technologies in the life and health industry as well as their far-reaching impact, so as to promote the research of protein structure and function, drug development and precision medicine, and bring new insights to researchers in related fields.

## 1 Introduction

Protein plays a key role in cell life activities and is the most abundant macromolecule in living organisms. It participates in a series of biological events, such as maintaining the structures and properties of organisms, catalyzing certain chemical reactions, transporting nutrients and metabolic wastes, providing a material basis for the body’s immune defense, participating in intracellular redox reaction, electron transfer, learning and memory.

The first person engaged in the purification of proteins was Edwin Joseph Cohn, an American biochemist, who purified serum protein (Labrou, 2021). In recent years, with the completion of genome sequencing of many species (Shendure and Lieberman Aiden, 2012; Beigh, 2016; Park and Kim, 2016; Ing-Simmons and Vaquerizas, 2019), researches in protein related fields have gradually become the focus of the biotechnology industry (Labrou, 2021), and many purification technologies have been gradually applied from protein and enzyme separation to drug synthesis, vaccine research and development, clinical detection, environmental analysis and biophysical measurement (Pfaunmiller et al., 2013). Therefore, high-quality protein samples are of great significance for the subsequent structure and function research and related products development (Tang et al., 2021). However, proteins have the characteristics of complex structure, easy to be affected by internal and external factors, easy to be degraded, etc. It is difficult and challenging to purify them without being contaminated by the host, maintaining structural integrity and biological activity.

As a downstream technology of the biological industry, the key to protein purification is to define the purification strategy and finally establish an optimal scheme through continuous optimization of purification conditions, that is, using the least steps to achieve the purity, concentration, the activity and the yield we need, which is crucial for subsequent researches (Lojewska et al., 2016; Owczarek et al., 2019; Schiermeyer, 2020). The purification strategy is mainly based on the unique physical and chemical properties and the three-dimensional structures of the target proteins, including the sequence and number of amino acids, the charge, polarity, and hydrophilicity/hydrophobicity of the polypeptide chains, the shape of the proteins, and the distribution of amino acid residues on the protein surface. The target proteins can be natural proteins from animals, plants and microorganisms, or recombinant proteins expressed by *E. coli*, yeast, mammalian cells and insect cells. Currently, recombinant proteins have become the main object of isolation and purification. Since different expression systems have their own advantages and disadvantages (Table 1), they should be selected according to the actual situation.

**TABLE 1:** Characteristics and applications of different expressed hosts.

Expression system	Advantages	Disadvantages	Applications
*E. coli*	*E. coli* is small and grows rapidly. It is comparatively easier genetic manipulation and fermentation process (Tripathi and Shrivastava, 2019), well genetically characterized (McElwain et al., 2022), simple cultivation conditions (Rosano and Ceccarelli, 2014), and a clear genetic background (Nagai et al., 2018). It has the potential of highly expressed proteins (Kesidis et al., 2020)	Target proteins are easy to form inclusion body and codon bias, which lacks the post-translational modification system (Liu et al., 2019) and endotoxin issues (Mamat et al., 2015), interfering with the expression of target proteins	The most commonly used prokaryotic expression system, widely used in the development of the vaccine (Shirley and Taha, 2018), the production of the hormone (Zielinski et al., 2019; Keshavarz et al., 2021), the synthesis of the important enzyme (Lu et al., 2018). The expressed protein is used for structural insights (Muller et al., 2022)
*Saccharomyces cerevisiae*	It has high expression level, rapid growth, easy culture, high tolerance to the environment (Tesfaw and Assefa, 2014), low culture costs (Duman-Scheel, 2019), and has a post-translational modification system (Baghban et al., 2019)	Recombinant proteins may accumulate excessively in cells, resulting in reducing the subsequent yield (Tomás-Gamisans, 2020). Intracellular post-translational modifications can lead to the production of hyperglycosylated proteins, which are immunogenic to the human body (Rasala and Mayfield, 2015; Xu et al., 2016). The replication plasmid YRp of *Saccharomyces cerevisiae* is unstable (Xie et al., 2018)	It can be used as an expression system for the inhibition of human dipeptidyl peptidase IV/CD26 (hDPPIV) (Zhang et al., 2016). It can produce hormones (Chigira et al., 2008), functional foods (Lahue et al., 2020), and biofuels (Boonchuay et al., 2021; Hong and Nielsen, 2012), and itself can also be used as probiotics
*Pichia pastoris*	As methanol is used as the sole carbon source and energy source, there are a methanol-regulated alcohol oxidase promoter (PAOX1) and a post-translational modification system, which has an efficient secretion mechanism (Juturu and Wu, 2018). It is highly similar to the mammalian cell expression system, but has lower culture costs and rapid expression than the mammalian cell expression system, more advantageous than *S. cerevisiae* (Karbalaei et al., 2020), and less endogenous secreted protein production to facilitate subsequent isolation and purification (Tachioka et al., 2016)	Glucose, glycerol, and ethanol severely inhibited promoter PAOX1 activity and affected exogenous protein expression (Turkanoglu Ozcelik et al., 2019). Methanol is flammable and toxic, which is detrimental to cell growth (Santoso et al., 2012)	It can express membrane proteins very well (Bill and Hedfalk, 2021). The production of anticancer drug (Liu et al., 2015), viral surface antigens (Gupta et al., 2020), antiviral proteins, important enzymes as well as viral subunit recombinant protein vaccines (Zhang et al., 2015; Chen et al., 2017), synthetic food-grade proteins and food additives (Bhataya et al., 2009; Zhang et al., 2021a)
Mammalian cell expression system	It can be naturally folded (Nigi et al., 2017) and has a highly similar post-translational modification system to human cells (Liu et al., 2022)	High culture costs, susceptible to animal viruses (Tripathi and Shrivastava, 2019), high growth conditions, long culture cycle (Tripathi and Shrivastava, 2019), low intracellular protein production	Expressing large and structurally complex recombinant proteins, such as secreted proteins and membrane proteins (Yeliseev et al., 2020). Produce drugs (Walsh, 2018), prepare monoclonal antibodies, produce important recombinant glycoproteins such as immunoglobulin G (IgG), growth factor, clotting factor VII, erythropoietin, and α-1 anti-trypsin (Nishida et al., 2017; Goh and Ng, 2018)
Insect baculovirus expression vector system	It is easy to cultivate and can express a large number of proteins. It has protein folding ability, and a better post-translational modification system (Kesidis et al., 2020)	Insect cells grew to a certain cell density and were infected with recombinant baculoviruses (Owczarek et al., 2019), thus the culture time is long, a large number of viruses are required. The costs of consumables and equipment required in the cultivation process is high (Kesidis et al., 2020), and the glycosylation modification system is not identical to the mammalian cells (Tripathi and Shrivastava, 2019)	It can express intracellular proteins, membrane proteins (Hardy et al., 2019), and secreted proteins very well. Prepare influenza vaccine (Trombetta et al., 2022) and virus-like particle (Chen et al., 2022), produce large protein complex (Zhai et al., 2019), produce highly efficient avian recombinant adeno-associated virus (Wang et al., 2017; Mi et al., 2021), produce cytoplasmic protein and secreted protein (Lemaitre et al., 2019), and use purified protein to develop detection reagents
Transgenic plants	It has low costs, relatively safe, low risk of contamination with animal pathogens, easy amplification and good stability. It is insensitive to metabolites, and has the ability to modify N-glycosylation (Dirisala et al., 2017; Lomonossoff and D’Aoust, 2016; Lojewska et al., 2016; Park and Wi, 2016; Buyel et al., 2017; Owczarek et al., 2019)	It is vulnerable to pesticides, herbicides and other pollution (Tripathi and Shrivastava, 2019)	Produce tumor immunotherapy agents (Chen et al., 2016; Hefferon, 2017). Develop the influenza vaccine (Loh et al., 2017; Owczarek et al., 2019). Synthesis of recombinases for disease therapy (Tekoah et al., 2015; Hefferon, 2017)
Transgenic animals	With a post-translational modification system and correct post-translational modification, the expressed target proteins have high yields and high product quality (Tripathi and Shrivastava, 2019)	Culture and subsequent purification costs are very high, long culture cycle, low scale-up capacity, vulnerable to virus, carcinogenic DNA and other contamination, and also vulnerable to ethical factors (Tripathi and Shrivastava, 2019)	Recombinant proteins are based on therapeutics, such as growth factors, monoclonal antibodies, vaccines and enzymes (Tripathi and Shrivastava, 2019)

**FIGURE 1:**
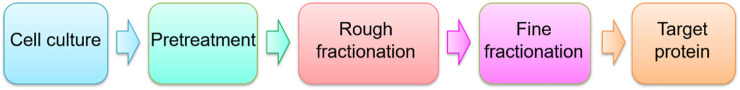
Basic steps of protein purification.

The complete protein isolation and purification steps generally include three steps: pretreatment, rough fractionation, and fine fractionation (Figure 1). In this review, the principles of common purification technologies and the recent advances made in this field are summarized, the applications of these technologies in life health industry and their far-reaching impact are discussed, and finally, the difficulties and challenges in the field of protein purification are prospected, in order to provide a variety of feasible solutions for the in-depth study of protein structure and function, and to bring new insights to readers in related fields.

## 2 Guidelines and strategies for protein purification

### 2.1 Identify the target of isolation and purification

Prior to purification, some basic properties of the target protein can be obtained through bioinformatics analysis softwares, such as the molecular weight (MW), isoelectric point (PI), solubility, molar extinction coefficient, stability, cysteine content, sensitivity to high concentration of salt ions or pH, secondary structure, hydrophilicity/hydrophobicity, sequence similarity to known proteins, susceptibility to oxidation, and potential post-translational modification sites, etc. Should we choose prokaryotic expression, eukaryotic cell expression, or insect expression system to express the target proteins? Can the correct post-translational modification of the target protein be ensured after expression? Which tag should be selected to facilitate overexpression and purification when constructing the vector? Which method should be used for cell disruption after expression, and whether the disruption process will cause the denaturation and structural change of the target protein? The purity, concentration, activity and yield of the final product should be determined to avoid over purification or failure to achieve the desired purity due to insufficient purification steps or resolution. All the above issues need to be considered comprehensively.

### 2.2 Reasonable selection of purification method

Select suitable purification methods according to the characteristics of the target protein and impurities, for example, choose to salt out, organic solvent precipitation, or choose centrifugation, electrophoresis, chromatography and other methods (Table 2). The purification scale should be determined according to the purpose of the target proteins, and the simple purification scheme should be used as far as possible to achieve the best effect. In addition, the size of the chromatographic column, the concentration of the protein obtained, whether it is necessary to maintain its activity and avoid unnecessary contaminants should also be considered. The additives should be used as little as possible, the impurities that damage the sample should be removed as early as possible, and the additional purification steps should be reduced.

**TABLE 2:** Separation basis and resolution of several purification methods (Labrou, 2021).

Separation mode	Separation basis	Resolving power
**Precipitate**		
Salting out	Solubility	Low
Organic solvent precipitation	Solubility	Low
Isoelectric point precipitation	Solubility, Charge properties	Low
Nonionic polymer precipitation	Solubility	Low
**Centrifugation**		
Differential centrifugation	Shape, Size	Low, Medium
Density gradient centrifugation	Size	Low, Medium
**Membrane separation**		
Dialysis	Size	Low, Medium
Ultrafiltration	Size/Shape	High
**Chromatography**		
Gel filtration chromatography	Size/Shape	High
Ion exchange chromatography	Charge	High
Hydrophobic interaction chromatography	Hydrophobicity	High
Affinity chromatography	Molecular recognition	High

### 2.3 Identify detection and analysis techniques

For protein solutions containing different components, the detection methods for target proteins are usually different. Different detection methods should be selected in different purification steps according to their sensitivity, precision, accuracy, etc., so as to evaluate the activity, purity and recovery of samples quickly and accurately.

### 2.4 Determine protein storage conditions

In order to improve the stability of the target protein, prevent microbial growth and maintain the protein activity, various surfactants, metal chelators, protease inhibitors, preservatives and reducing agents (sh-mercaptoethanol or DTT) can usually be added to the buffer solution. In addition, under the premise of ensuring the activity of the target protein, it should be stored at −70°C, and repeated freezing and thawing should be avoided.

## 3 Cell culture

Recombinant protein synthesis is the process of producing proteins in prokaryotic microorganisms or eukaryotic cells using recombinant DNA technology, and it is an important branch of the emerging synthetic biology. It is because of the development of recombinant DNA technology that people can produce a large number of protein products for research or treatment. Historical experience has continuously proved that the manufacture of a key protein molecule can quickly promote the rapid development of related fields, such as recombinant human insulin and recombinant human growth factors.

**FIGURE 2:**
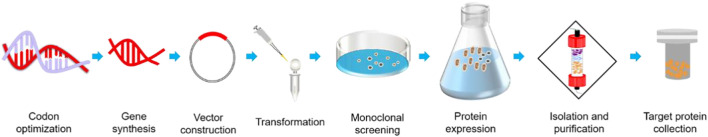
Cultivation and purification process of recombinant protein *Escherichia coli* expression system.

At present, the biggest bottleneck of recombinant protein manufacturing technology lies in “manufacturing”. The first difficulty is whether the target proteins can be expressed in large quantities by microorganisms or cells, and the second is how to obtain high-quality and active proteins through a series of processes and purification. Nowadays, expression systems such as *E. coli*, yeast, mammalian cells, insect cells, transgenic plants and transgenic animals are often used to obtain recombinant proteins. Here we take *E. coli* as an example to introduce the expression process of the target proteins. Firstly, the target gene was determined and suitable vectors (Table 3) and competent cells (Table 4) were selected, then the target gene was linked to an empty vector to construct a recombinant expression vector, and then the recombinant vector was transformed into host cells for culture. When OD_600_ reaches 0.4–0.6, an appropriate proportion of inducer was added to induce protein expression at a certain temperature, and finally the thalli were collected for purification (Figure 2). As an expression host, *E. coli* has become one of the best hosts for the production of recombinant proteins due to its rapid reproduction, low costs, and rapid expression of a large amount of target proteins (Rosano and Ceccarelli, 2014). In order to avoid the influence of lack of post-translational modification systems, inclusion body formation, frameshift mutations and endotoxin production on subsequent purification, several means such as changing host, vector and target gene sequence constantly, optimizing culture conditions, and co-expressing target proteins with molecular chaperones are adopted by researchers to increase protein solubility and expression (Gopal and Kumar, 2013). For the problems encountered in the expression process, a series of effective solutions have been developed. For example, T7RNAP expression activity (Li Z. J. et al., 2022; Chee et al., 2022) or growth decoupling system (Li et al., 2016; Darlington et al., 2018) can be specifically regulated when expressing toxic proteins, but this method is not universally applicable. Zhang et al. (2022) developed a dynamic equilibrium system that can achieve the overexpression of basic growth-related genes (rRNA, RNAP core enzyme, sigma factor), accurately predict and express key proteins using an ec_iECBD_1354 enzyme constraint model, and dynamically regulate the expression intensity of key growth-related proteins based on a load-driven promoter. This system alleviates the host burden effect, improves the production of recombinant proteins, and is helpful to efficiently develop expression hosts based on the properties of target proteins. However, the *E. coli* expression system lacks post-translational modifications, which is not conducive to the expression of eukaryotic proteins and many enzymes (Gupta et al., 2019), and increases the subsequent purification processes. Currently, the modified *E. coli* expression system can achieve the simple glycosylation modification of proteins (Gupta and Shukla, 2016), but it still needs to be further optimized. Some rare codons exist in protein-coding genes of higher animals, but these rare codons are not common in *E. coli*. Therefore, the expression of these proteins by *E. coli* expression system may lead to the reduction of target protein expression or premature termination of protein translation (Owczarek et al., 2019). In view of this, several online analysis softwares have been developed to detect the presence of rare codons in the gene sequence, and the expression of the target proteins can be improved through codon optimization (Gupta et al., 2019; Rosano et al., 2019). In addition, introducing short-term heat shock before induction (Oganesyan et al., 2007), adding D-sorbitol, glycerol, ethanol, NaCl (Diamant et al., 2001; Bowden et al., 1991; Oganesyan et al., 2007) and other chemical additives and/or target protein cofactors (Bushmarina et al., 2006; Rosano and Ceccarelli, 2014), glucose, lactose (Tahara et al., 2021) to the medium to change the culture conditions, and reduce the culture temperature (Carere et al., 2018; Wang et al., 2019) and IPTG concentration (Jhamb and Sahoo, 2012; Sina et al., 2015) are also effective ways to induce the high expression of the target proteins. Lactose operon *lac* is an inducible gene expression element, and 0.5–1 M IPTG is generally used to prevent LacI (repressor) from binding to the operator gene (Donovan et al., 1996) to achieve the expression of the target gene. In addition, to reduce the formation of inclusion bodies, coexpression of target proteins with molecular chaperones (de Marco et al., 2007; Jhamb and Sahoo, 2012; Rosano and Ceccarelli, 2014), use of weak promoters and low-copy plasmids (Kaur et al., 2018), adding fusion tags to the N-and/or C-terminus of expression vectors (Liu et al., 2019; Ruan et al., 2020), and adding signal peptides to import the target proteins into the periplasmic region (Dow et al., 2015; Vargas-Cortez et al., 2017) are usually adopted.

**TABLE 3:** Commonly used plasmids and characteristics of the *E. coli* expression system (Kesidis et al., 2020).

Plasmid series	Promoter	Derivant	Condition of culture (°C)	Repressible system	Prokaryotic resistance	Replicon	Cloned strain
pET	T7	IPTG	37	LacI	Amp, Kan,Str	pBR322	DH5α
pGEX	Tac	IPTG	37	LacI	Amp	pBR322	DH5α
pQE	T5	IPTG	37	LacI	Amp, Kan	pBR322	DH5α
pBAD	araBAD/BAD	Arabinose	37	araC	Amp,Chl, Spe,Apr	pBR322, p15A, and pSC101	DH5α
pMAL	Tac	IPTG	37	LacI	Amp	pBR322	DH5α
pASK-IBA	Tet	Anhydrotetracycline	37	Tet-repressor	Chl, Amp	pBR322	DH5α
pRSET	T7	IPTG	37	LacI	Amp	pBR322	DH5α

**TABLE 4:** *E. coli* BL21 and its derived host for recombinant protein expression.

Strain name	Important features	Application
BL21	*E. coli* polymerase but no T7 RNA polymerase	It is mainly suitable for the expression of non-toxic proteins, and it can be used for the protein expression of plasmids (such as pGEX and pMAL)
BL21 (DE3)	The λ phage DE3 region containing T7 phage RNA polymerase is integrated on chromosome BL21 allowed the simultaneous expression of both T7 RNA polymerase and *E. coli* RNA polymerase. (Ivanov et al., 1993)	For the efficient expression of the genes cloned in the expression vectors containing the phage T7 promoter (such as the pET series), and for the protein expression of the plasmids (such as pGEX and pMAL)
BL21 (DE3) pLysS	Carrying the pLysS plasmid, having chloramphenicol resistance, and containing genes expressing T7 lysozyme. T7 lysozyme can reduce the background expression level of the target gene, but does not interfere with IPTG induced expression	Suitable for the expression of toxic proteins and non-toxic proteins
BL21 (AI)	It has tetracycline resistance (Bhawsinghka et al., 2020), lacks Lon protease and OmpT extrtramembrane protease. It can effectively prevent the degradation of heterologous proteins in *E. coli in vivo*	Suitable for any T7 promoter-based expression vectors. Widely used for the high-level expression of toxic recombinant proteins (Bhawsinghka et al., 2020)
BL21 Star (DE3)	It contains *E. coli* RNA polymerase, has chloramphenicol resistance, and contains the rne131 mutation, which reduces the accumulation of endogenous RNase and enhances mRNA stability of intracellular mRNA in the stain (Jiang et al., 2021), thereby it increases the expression level of heterologous proteins	Suitable for the high-level protein expression of the T7 promoter expression vector (such as the pET series) and the non-T7 promoter expression vector (such as pGEX and pMAL), it is not suitable for the expression of toxic proteins (Jiang et al., 2021)
BL21 Star (DE3) pLysS	Containing the *E. coli* RNA polymerase, carrying the pLysS plasmid, having chloramphenicol resistance, and containing the rne131 mutation, enhances the cellular stability of the mRNA within the strain, thus increasing the expression level of the heterologous protein. Lower the background expression level of the target gene, but did not interfere with the IPTG-induced expression	It is suitable for high-level expression of protein expression in T7 promoter expression vectors (such as the pET series) and non-T7 promoter expression vectors (such as pGEX and pMAL)
BL21-CodonPlus (DE3)-RIPL	It is absent of the Lon protease and the OmpT protease, thus reducing the degradation of the recombinant proteins and enhancing the expression levels of foreign genes, In particular, the expression levels of AT-or GC-rich eukaryotic genes in the prokaryotic system. It can simultaneously express T7 RNA polymerase and *E. coli* RNA polymerase, and has resistance to tetracycline, chloramphenicol, streptomycin, and spectacular mycin	It can be used for the protein expression of plasmids (such as the pET series, pGEX, and pMAL)

## 4 Pretreatment

Before purification, the sample should be pretreated to release the total proteins from the tissue cells while maintaining the activity of the target proteins. Since different tissues and cells have different structures, different materials need to be pretreated by different methods. Commonly used pretreatment methods include mechanical method and non-mechanical method, the former includes high-speed bead milling, high-pressure homogenization and ultrasonic crushing, etc., while the latter includes osmotic pressure impact, freeze-thaw crushing, enzymatic hydrolysis and chemical crushing, etc. For example, connective tissue and adipose tissue should be removed by grinding, ultrasonic crushing (Tang et al., 2015) and centrifugation before the purification of animal materials. The seed materials should be shelled or even peeled to avoid the pollution of tannins and other substances, especially the oil seeds should be degreased with low boiling organic solvents such as ether. Plant tissues contain a wide variety of compounds, such as phenols, lipids, pigments, organic acids, carbohydrates, etc., which greatly interfere with protein extraction and proteomic analysis (Wang et al., 2008). In the purification of natural plant proteins, appropriate parts should be selected first for sampling, and then the cell wall and other components should be broken by mechanical or non-mechanical methods (Kielkopf et al., 2021) to obtain more cell lysates (Hunt, 2005), so as to ensure the smooth progress of the subsequent purification process. The purification of recombinant proteins produced by genetic engineering technology also requires pretreatment. The *E. coli* expression hosts are often collected by centrifugation at low temperature and broken by ultrasound. In addition, due to the mild and highly efficient action conditions of the enzyme, selecting a suitable enzyme can effectively decompose the plant or bacterial cell wall to achieve a better purification effect. Therefore, enzymatic hydrolysis has also been widely used in the process of sample pretreatment in recent years. When extracting oil crop proteins, the use of Viscozyme can fully degrade the cell wall structure and promote the release of oil and protein in peanut cells (Liu et al., 2020a). Penha et al. (2020) using Viscozyme L under the condition of 50°C can improve the recovery rate of soybean protein from 42% to 83%, the recovery rate of isoflavones from 59% to 93%, and also reduce the residue of soybean dregs by 85%, so the utilization rate of raw materials was better improved. Enzymatic hydrolysis can also reduce the sensitization of food. Liang et al. (2021) found that Alcalase, Protamex and Flavourzyme could decompose the sensitizing proteins such as casein, β-lactoglobulin and α-lactoferrin in milk, which not only reduced the sensitization of milk but also improved the content of free amino acids and nutritional quality. Therefore, the enzymatic hydrolysis can also be used to produce hypoallergenic dairy products to avoid allergic events. The above pretreatment methods can also be used as auxiliary means in the subsequent rough fractionation and fine fractionation, which can not only improve the extraction efficiency of the target proteins, but also reduce the use of some solvents (Li et al., 2021).

## 5 Rough fractionation

Protein extracts after pretreatment usually contains impurities such as cell debris, aggregates, nucleic acids, lipids and polysaccharides (Royce et al., 2018), it is necessary to select a set of appropriate methods to separate the target proteins from the impurities, while avoiding the degeneration and degradation of the target proteins.

### 5.1 Precipitation method

Precipitation method is based on the difference of solubility between proteins. By adding an appropriate precipitant to the protein extracts, the solubility of the target proteins can be changed to make it precipitate or aggregate, so as to achieve the effect of separation. Precipitation method can obtain a large amount of target proteins from cell extracts quickly and in batches (Matulis, 2016), which is a suitable choice for rough fractionation. The commonly used precipitation methods include salting out, isoelectric point precipitation and organic solvent fractionation.

#### 5.1.1 Salting out precipitation

The solubility of protein is susceptible to the influence of ionic strength in solution. At low salt concentrations, the solubility of proteins will increase with the increase of salt concentrations in the solvent. This phenomenon is called salting in, and the proteins at this time still maintains the folded conformation and stability. If the salt concentration continues to increase, the hydration force of the solution will be enhanced, leading to the destruction of the hydration film on the surface of the proteins, resulting in the reduction of protein solubility, and then aggregation or precipitation. This phenomenon that the solubility of proteins decreases with the increase of salt concentrations is called salting out (Duong-Ly and Gabelli, 2014). The salting out method has the advantages of safety, mild action conditions, maintaining the biological activity of the target protein, low cost and simple operation. However, a large amount of salting-out agent is often left in the sediment, and some contaminates will also precipitate along with the target proteins. Therefore, desalination treatment is also an important part of the salting out precipitation method. Commonly used neutral inorganic salts are ammonium sulfate, sodium sulfate, magnesium sulfate and potassium phosphate. Ammonium sulfate has the advantages of good solubility, low price, easy preparation of high purity products, stability of protein structure and so on. It is the most commonly used neutral inorganic salt in salting out, especially for the purification of acidic proteins. In addition, ammonium sulfate can also be used with other precipitants to achieve better precipitation effect. Ovotransferrin has the functions of anti-oxidation, antibacterial and promoting iron absorption (Rathnapala et al., 2021). Abeyrathne et al. (2013) separated ovotransferrin from egg white by combining ammonium sulfate precipitation with critic acid. This process does not introduce highly polluting chemical reagents, so the ovotransferrin isolated by this method can be used in food and drug production after ultrafiltration desalination. In practical research, the optimal salt concentration of protein precipitation is usually determined by gradually increasing the salt concentration to separate the target proteins one by one under certain temperature and pH conditions. Hazim Abdul Hameed and Hussein Ali (2021) reported that 60% ammonium sulfate was the optimal ratio for rough fractionation when purifying the extracellular L-glutamate oxidase of *Streptomyces*, and the specific activity of the target protein could reach 8.25 U/mg. In addition, the ammonium sulfate salting out precipitation method can also be used for the isolation and purification of special DNA structures, such as the purification of DNA origami nanostructures reported by Hanke et al. (2022).

#### 5.1.2 Organic solvent precipitation

Organic solvent precipitation is another commonly used method for rough fractionation of proteins, the principle of organic solvent precipitation is that multifold organic solvent is added to the extract of biological macromolecules such as proteins to reduce the dielectric constant of the solution and increase the interaction between protein molecules, so that the solubility of proteins is significantly reduced and eventually aggregation or precipitation occurs. This method is conducive to the precipitation of high molecular weight proteins, while low molecular weight proteins or peptides are not easy to aggregate in organic solvents and remain in the supernatant (Baghalabadi and Doucette, 2020). Different organic solvents usually differ in their abilities to precipitate proteins, and common organic precipitants include acetone, isopropanol, ethanol, and methanol.

Organic solvents are usually volatile, so the residual organic solvents in the target proteins can be easily and quickly removed, and some organic solvents themselves can also be used as protein bactericides. As a commonly used organic solvent, acetone can greatly reduce protein degradation, have little impact on protein activity, and avoid contamination of impurities such as salt and polyphenol (Shaw and Riederer, 2003). When using the organic solvent precipitation to purify proteins on a large scale, it is necessary to first determine the optimal solvent concentration of the target proteins and the optimal volume ratio of protein extract to precipitant. The organic solvent selected as the precipitant should not have the ability to dissolve the target proteins, so the precipitant can be used to dissolve the soluble substances first, and then select an appropriate way to further extract the target proteins and other insoluble substances remaining in the precipitates (Wang et al., 2008; Wu et al., 2014). However, some organic solvents can destroy the hydrogen bonds in proteins and change the spatial structure of target proteins. For example, organic solvents such as ethanol release heat when mixed with water, which is easy to cause protein denaturation and inactivation. Furthermore, the precipitation process needs to consume a large amount of organic solvents and most of them are toxic and flammable, the operation is complex and the costs are high, the process needs to be carried out at low temperature, and the recovery rate is also lower than that of the salting-out method, all these make this method has certain limitations. However, in proteomic analysis, organic solvent precipitation can selectively deplete high molecular weight proteins or enrich low molecular weight proteins in soluble components, increase the detection coverage of small molecular weight proteins, and help improve the accuracy of proteomic analysis (Periasamy et al., 2021; Nickerson et al., 2022).

#### 5.1.3 Isoelectric point precipitation

The principle of isoelectric point precipitation is based on the fact that different proteins have different isoelectric points. When the pH value of proteins extract reaches the isoelectric point of the target protein, the net charge of the target protein is zero, the solubility is minimum, and the conformation is in the most compact state (Matulis, 2016), and no swimming occurs in the electric field, so as to achieve the purpose of separation. The isoelectric point precipitation is easy to operate and has various pH adjustment methods. However, this method also has defects. For example, when the pH reaches the isoelectric point of the target protein, it still has a certain solubility, resulting in incomplete precipitation. In addition, the inorganic acid/base (hydrochloric acid and sodium hydroxide) introduced in the adjustment of pH is easy to cause irreversible denaturation of the target proteins, and the isoelectric points of many proteins are relatively close, so the effect of isoelectric point precipitation alone is not ideal, with poor resolution and low purity, and generally only used for the preliminary purification of proteins. In order to solve the above problems, isoelectric point precipitation is often used together with other methods. For example, Watanabe et al. (2015) used isoelectric point precipitation combined with electrolytic water treatment technology to purify proteins from rice bran, which can not only significantly improve the purity and extraction efficiency of proteins, but also recover phosphorus containing compounds. Li et al. (2021) used ultrasonic assisted alkaline electrolyzed water to extract proteins from Euphausia superba, which can not only reduce the consumption of NaOH, but also maintain the activity of proteins and improve the yield of krill proteins.

#### 5.1.4 Other precipitation methods

Nonionic polymer precipitation was first used to extract immunoglobulin. In recent years, it has been widely used in the isolation and purification of proteins, polypeptides, nucleic acids and enzymes (Wang et al., 2008). The polymers include dextran, NPEP, and polyethylene glycol (PEG) with different molecular weights.

The commonly used polymer PEG is an inert substance with high stability to proteins and can be stored at −20°C for a long time. PEG is generally harmless to the human body and is widely used in cosmetics and medical products (Geng et al., 2019), but some people may develop systemic allergic reactions when using these products (Sellaturay et al., 2021). PEG combined with other forms of precipitant can improve the purification efficiency and reduce the consumption of some substances. Aqueous two-phase systems (ATPS), for example, have emerged as an alternative technology for protein recovery and concentration. Menegotto et al. (2021) established the maximum recovery condition of A. platensis protein using 16% sodium citrate and 18% PEG (1500 Da), and achieved a purification factor of 1.02 and a protein recovery rate of 75%. Geng et al. (2019) used 15% PEG to separate ovalbumin, ovomucin and ovotransferrin at 10°C, pH6.5 and 100 mM NaCl, then obtained a purity of 95.1% ovalbumin by isoelectric precipitation (pH4.5, 4°C) combined with HPLC purification, and the recovery rate was 46.4%. Ovalbumin can be extracted from several kilograms of egg white within 2–3 h. Furthermore, PEG is easier to extract LDL from egg yolk than ammonium sulfate (Wang et al., 2018).

### 5.2 Centrifugation method

The principle of centrifugation is that when an object moves in a circle around a central axis, the moving object is subjected to centrifugal force, under the action of centrifugal force to achieve the purpose of protein separation. Centrifugation is also a suitable choice for rough fractionation, which can be used as a rough fractionation scheme alone or as a key step in multi-step purification. For example, after precipitation or mechanical crushing, more precipitates can be gathered together by centrifugation to achieve a better separation effect. The commonly used centrifugation methods in protein purification are differential centrifugation and density gradient centrifugation.

#### 5.2.1 Differential centrifugation

**FIGURE 3:**
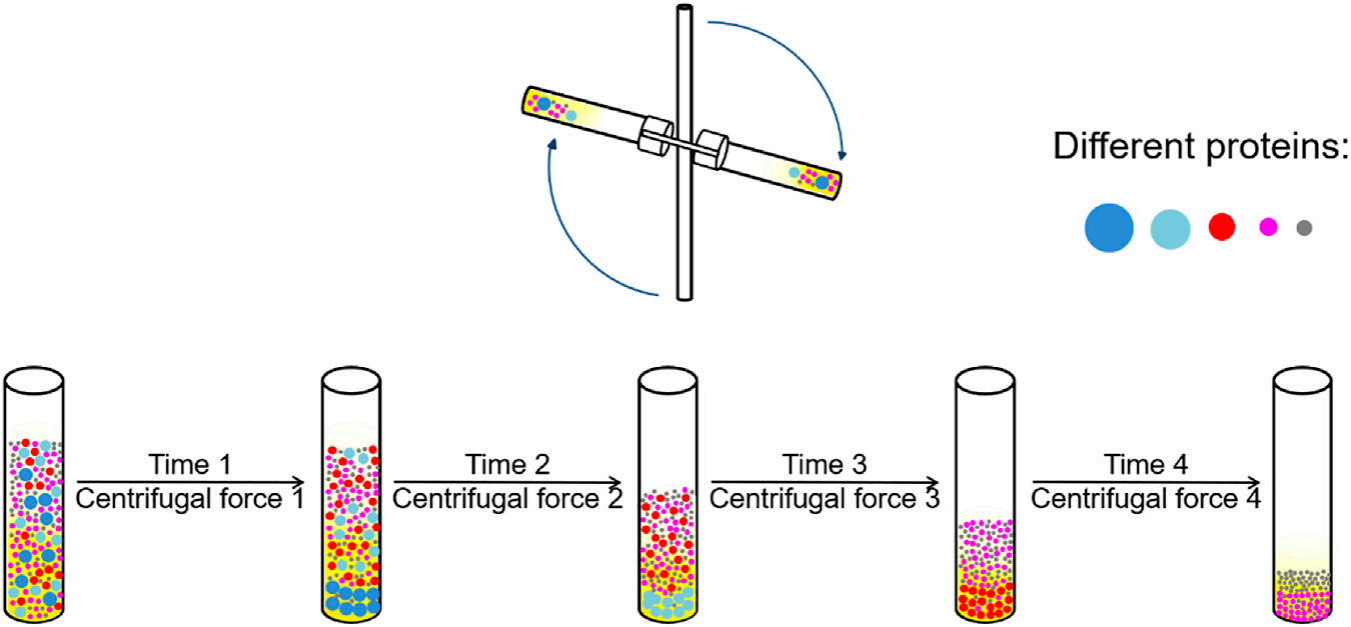
Differential centrifugation process. Different protein components were precipitated different centrifuge tubes with different centrifugal force and times.

Differential centrifugation is based on the difference in sedimentation velocity of proteins with different sizes. The method achieves the effect of separation by continuously increasing the relative centrifugal force, controlling the centrifugation time, and conducting multiple centrifugations (Figure 3). Muthunayake et al. (2020) used differential centrifugation to enrich Bacterial Ribonucleoprotein Bodies (BR bodies) from *Caulobacter crescentus*. Ma et al. (2020) isolated the apoptotic bodies by differential centrifugation after inducing the apoptosis of osteoclasts. Differential centrifugation can obtain different cells more quickly than the conventional washing method during the isolation of mouse bone marrow cells, and it does not affect the cell viability and the distribution of hematopoietic cell populations (Heib et al., 2021). The advantage of differential centrifugation is that it is relatively convenient to separate multiple samples simultaneously. However, this method requires repeated centrifugation, which is more complex. In addition, the precipitate needs to be washed, dissolved and re precipitated for many times during the purification process, which is easy to cause sample loss and yield reduction.

#### 5.2.2 Density gradient centrifugation

**FIGURE 4:**
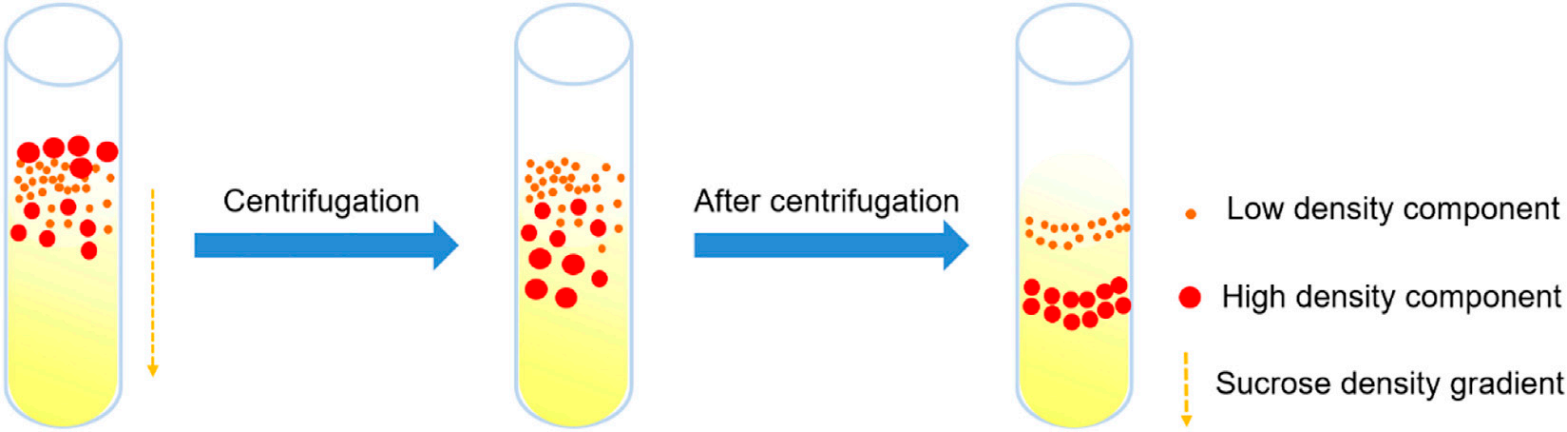
Density gradient centrifugation process.

Density gradient centrifugation is also called zonal centrifugation. The protein samples to be separated were placed on the surface of density gradient formed by the medium (sucrose, cesium chloride, etc.) with gradually increasing density and high solubility, and the proteins of different shapes and sizes were separated by centrifugal force to form different settling zones at different settling speeds (Figure 4). This method can separate multiple components in mixed samples with high resolution and low cost. At present, it has been widely used for the isolation and purification of cells, organelles, viruses, bacteria, nucleic acids, proteins, etc. For example, the inner and outer membrane of liver tissue can be separated by sucrose density gradient centrifugation (Lin et al., 2010), and mitochondria after differential centrifugation can also be further refined purified (Clayton and Shadel, 2014). Szelechowski et al. (2013) purified adenoviruses and bacteriophages by cesium chloride density gradient centrifugation. Nasukawa et al. (2017) reduced the centrifugal force from 100,000g to 40,000 g and increased the centrifugation time from 1 to 2 h under the condition of 4°C, and completed the phage purification by non ultracentrifugation, realizing the virus purification without ultracentrifuge. In the final step of bacterial ribosome purification, polysomes were purified for *in vitro* cell-free translation systems by centrifugation of samples containing 70S monomers, ribosomal subunits, and polysomes through a linear sucrose gradient of 7%–30% (w/w) (Rivera et al., 2015). Wichmann et al. (2021) used OptiPrep density gradient centrifugation to isolate a higher proportion of bacteria from the complex matrix with low microbial load, confirming the compatibility of this method with Raman spectroscopy, and the combination of the two methods can improve the accuracy of bacterial infection diagnosis.

### 5.3 Dialysis method

Dialysis is a method to separate colloidal substances such as proteins from other small molecular substances including inorganic salt ions and reducing agents based on the principle that protein and other macromolecular particles are large, have colloidal properties, and cannot freely pass through the semi-permeable membrane (Echave et al., 2021). The activity of purified protease can be improved by attaching specific substances targeting the target protease to the semi permeable membrane (Labus et al., 2020). In order to improve the recovery rate of the target proteins during dialysis, factors such as buffer exchange time, the design of dialysis system, and the chemical and morphological characteristics of dialysis membrane should be considered. In terms of buffer exchange time, the increasing temperature can accelerate the molecular movement and increase the intrinsic diffusion of proteins across the semi-permeable membrane to reduce dialysis time, but increasing temperature may also have adverse effects on protein activity. Therefore, the maximum temperature at which protein activity can be maintained should be determined in advance when using dialysis for protein purification (Burgess, 2016). Dialysis is also commonly used for desalting treatment after the end of salting out. When the protein solution is in a high concentration and high salinity state, dialysis is usually the best method to remove ammonium sulfate from the sample (Duong-Ly and Gabelli, 2014). Phycocyanin has antioxidant, anticancer, liver and kidney protection effects (Piovan et al., 2022), and has been widely used in the production of drugs and nutritional products in recent years. Khazi et al. (2020) pretreated *Cyanobacteria* to obtain the crude extract of phycocyanin. Ammonium sulfate was added into the crude extract in sections, and the supernatant was removed by centrifugation after full stirring, then the phycocyanin was resuspended and dialyzed overnight to achieve further purification effect.

### 5.4 Ultrafiltration method

Ultrafiltration is also a method based on membrane separation, which can achieve the effect of concentration and is also a common way of desalination (Thammasena et al., 2020). The performance of ultrafiltration membrane is the key to the success of protein separation. Most ultrafiltration membranes consist of a sturdy scaffold structure with a very thin polymer layer attached to it. Cellulose acetate can be used for filtration, gas separation, adsorption and ion exchange (Vatanpour et al., 2022) due to its advantages of film-forming, good chemical activity (Ma et al., 2022), high stability (Muhmed et al., 2019) and hydrophilicity, environmental protection, and appropriate costs (Sharma et al., 2020; Syamani, 2020). Although the production technology of membranes used for ultrafiltration has not changed in the past few decades, the control technology of thin layer pore size distribution, membrane morphology and membrane modification has been significantly improved. It is this thin layer that provides the properties required for a selective permeability membrane and determines the flow resistance (Burgess, 2016). The new dopamine modified cellulose acetate ultrafiltration membrane is a relatively advanced filter membrane. The use of Dopamine-modified cellulose acetate overcome the trade-off between permeability and selectivity of conventional cellulose acetate membranes to some extent, possess good antifouling capability and long-term stability, and intercept effectively the target protein as well as improve the water permeability (Ma et al., 2022). In order to avoid the accumulation of intercepted proteins on the membrane surface, tangential flow filtration can be used (Busatto et al., 2018). The principle is that the protein solution is driven by the pump to flow along the direction tangent to the membrane surface. The pressure difference formed on the membrane makes part of the liquid pass through the membrane, while the other part of the liquid flows tangentially through the membrane surface to wash away the intercepted protein molecules. The currently developed one-way tangential flow ultrafiltration technique enables the continuous ultrafiltration of mAb by increasing the surface area and the residence time (Thakur et al., 2022). Ultrafiltration is also applied to the treatment of diseases such as acute heart failure (Costanzo, 2019), the removal of pathogens from seawater (Cordier et al., 2020), the purification of the vaccine product (Emami et al., 2019) and recombinant ferritin (Palombarini et al., 2019), and the treatment of galvanized waste-water (Oztel et al., 2020).

To sum up, the rough fractionation methods are relatively simple and have a large processing flux. They can not only remove a large number of impurities, but also concentrate the protein solution to achieve partial purification, while maintaining a high recovery rate (Mazi et al., 2016). Among the above rough fractionation techniques, PEG precipitation, ultrafiltration, differential centrifugation and other methods can achieve the concentration of viruses in wastewater (Dumke et al., 2021). During the SARS-CoV-2 pandemic, many countries used these methods to enrich and detect viruses in wastewater, thereby boosting epidemiological surveillance and playing an early warning effect (Ahmed et al., 2020; Medema et al., 2020; Ai et al., 2021; Hillary et al., 2021). However, the effect of rough fractionation is poor, so in some important structural and functional analysis studies, it is necessary to combine more refined fractionation methods to obtain the target proteins with higher purity.

## 6 Fine fractionation

After a series of pretreatment and rough fractionation, the volume of the sample was reduced and most of the impurity proteins were removed. The purpose of fine fractionation is to separate the target proteins from some proteins of similar size and physical and chemical properties to obtain higher purity, so as to meet the needs of different research fields. At the same time, higher resolution and stronger specificity are required in the process of fine fractionation.

### 6.1 Chromatography

Chromatography is based on the difference in physical and chemical properties of different substances (Scopes, 2001). It has been used to extract plant pigments as early as the beginning of the 20th century. At present, chromatography has been widely used in the separation of proteins, nucleic acids, polysaccharides, peptides and other biological macromolecules. Chromatography can be divided into adsorption chromatography and non-adsorption chromatography according to whether the sample is bound to the filler. The former mainly includes ion exchange chromatography, hydrophobic chromatography, affinity chromatography and reverse phase chromatography, while the latter mainly includes gel filtration chromatography. Several commonly used chromatographic techniques are introduced as follows. (Table 5).

**TABLE 5:** Comparison of common chromatographic separation methods.

Types	Gel filtration chromatography	Ion exchange chromatography	Hydrophobic chromatography	Affinity chromatography
Characteristic				
Separation mechanism	Size	Charge	Hydrophobicity	Specific affinity
Selectivity	Secondary	High	Medium/high	Very high
Load	Low	High	High	High
Purification rate	Medium/low	High	High	High
Biocompatibility	Very nice	Good	Medium/good	Good
Purified protein yield	High	High	Medium/high	High
Capture concentration phase	+	+++	+++	+++
Intermediate purification stage	+	+++	+++	+++
Refining and purification stage	+++	+++	+	++

#### 6.1.1 Gel filtration chromatography

**FIGURE 5:**
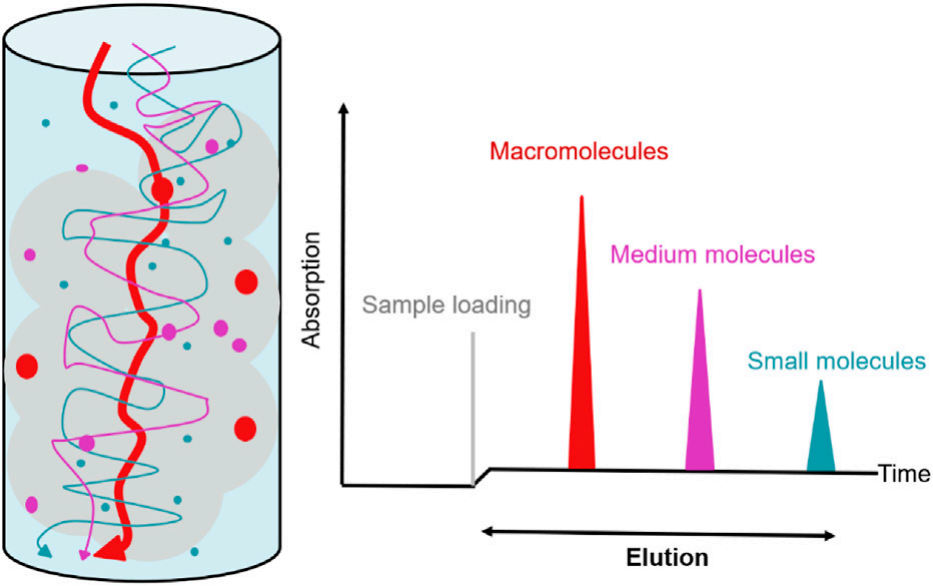
Principle of gel filtration chromatography. Collect effluents at different times according to different target protein sizes.

Gel filtration chromatography, also known as molecular sieve chromatography, is one of the most effective methods developed in the 1960s to separate and purify different proteins based on molecular size, and is also the gold standard for separating protein polymers from their monomers (London et al., 2014). In the elution process, proteins with high molecular weight first flow out along the gap between the gel particles, while proteins with low molecular weight can enter the gel mesh, resulting in flow obstruction and slow outflow. The required proteins can be collected according to the time sequence of elution **(** Figure 5 **)**. The matrix for gel filtration can be composed of a variety of materials, including simple substances such as dextran (Sephadex™ Series), agarose (Sepharose™ Series) and polyacrylamide (bio-Gel Series) or mixtures composed of dextran polyacrylamide (Sephacryl™ Series) or dextran-agarose (Superdex™ Series). Gel filtration chromatography, which has the advantages of simple operation, rapid separation without affecting biological activity, has been widely used for the isolation and purification of proteins or peptides (Gao et al., 2016). At the same time, the dextran gel can also be used for desalination after salting out. However, the gel filtration chromatography also has certain defects. The chromatography column is longer than other separation columns, so the flow rate is slower, the elution time is longer, and higher column pressure and more fillers are required. Gel filtration chromatography is often used in combination with other chromatography methods (Li Z. et al., 2022). To further investigate the effect of D614G substitution on the structure of SARS-CoV-2 spike protein, Zhang et al. (2021) purified the SARS- CoV-2 spike protein carrying D614 or G614 with detergent DDM, further purified it through gel filtration chromatography and used it for structural analysis, providing a structural basis for the development of new coronavirus vaccine. Ye et al. (2022) used 70% methanol as elution buffer, and obtained high concentrations of isoorientin and four flavone C-glycosides from bamboo leaf flavonoid by gel filtration chromatography. Gel filtration chromatography can also be used in the last step of purification of low molecular weight hyaluronic acid (Karami et al., 2021). Cao et al. (2022) used TALON IMAC metal chelation chromatography and gel filtration chromatography to obtain pure serotonin 2A receptor (5-HT2AR) for research on the treatment of depression. Zhang Y. et al. (2021) obtained high-purity NMDA receptors by combined use of Strep-Tactin affinity chromatography and gel filtration chromatography (Superose six Increase), and the analysis of its three-dimensional structure by cryo-EM provided new insights for the development of antidepressants. Moreover, gel filtration chromatography also plays an important role in medical examination and is the gold standard for the detection of macroprolactin (Vilar et al., 2019). When the recovery rate of thyroid stimulating hormone (TSH) does not decrease, the gel filtration chromatography can be used to detect the presence of macro-TSH as the evaluation index of normal thyroid function (Fukushita et al., 2021).

Gel filtration chromatography is suitable for the separation of proteins with larger or smaller molecular weight. When the molecular weight of proteins in the sample is moderate, the purity of the target proteins obtained is relatively low. When the molecular weight of proteins is 25% different from each other, they can be completely separated by a single gel column. In addition, the viscosity of the sample should not be too high, otherwise the mass transfer resistance will increase, and the gel particles will sometimes have non-specific adsorption, which is easy to block the column.

#### 6.1.2 Ion exchange chromatography

**FIGURE 6:**
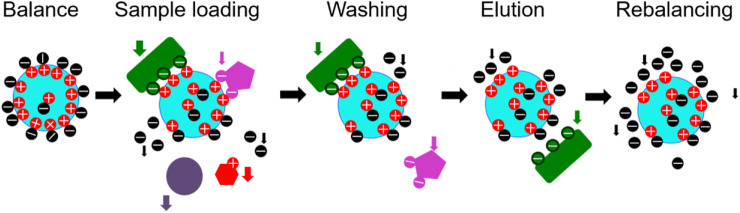
Principle of ion exchange chromatography.

Ion exchange chromatography uses ion exchanger as stationary phase to separate and purify according to the difference in reversible binding strength between component ions in the mobile phase and equilibrium ions on the exchanger. Ion exchangers are made by introducing several dissociable groups (active groups) into an insoluble polymer substance (the parent body). It is the many covalently bound charged groups and convertible ions fixed to the parent body that play a key role in the chromatography process. The interaction strength between the proteins and ion exchangers changes with the buffer salt concentration or pH, and the protein is eluted according to the binding strength, so as to achieve the purpose of separation and purification. According to the properties of active groups, ion exchangers can be divided into cation exchangers and anion exchangers. According to the difference of parent, ion exchangers can be divided into ion exchange resin, ion exchange cellulose, and ion exchange gel. The basic steps of ion exchange chromatography include: balance, loading, washing, elution and rebalance **(** Figure 6 **)**. Generally, the sample loading is completed under the condition of low salt ion concentration, and elution is carried out with high concentration salt ion buffer solution. Factors affecting the interaction between ion exchanger and protein include the charge of protein and ion exchanger, dielectric constant of medium, competition of other ions for ion exchanger and charged group of protein, charge distribution on protein surface, properties, temperature, additives of special ions in solvent, and non-electrostatic interaction and hydrogen bond between protein and ion exchanger (Evert and Irwin, 2011).

Ion exchange chromatography has the advantages of moderate costs, high resolution, easy expansion, and large-scale purification. Low concentration protein and polynucleotide solution can be rapidly concentrated to achieve the purification effect (Evert and Irwin, 2011). At present, it has been automated and has become the most functional and widely used one among all chromatography technologies. However, even under the most accurate conditions, pure target protein cannot be obtained by ion exchange alone, so it is necessary to combine centrifugation, salting out and other steps to achieve fine purification. Antibacterial proteins produced by plant tissues are effectively effective against microbial invasion (Gonzalez-Lamothe et al., 2009). Radhakrishnan et al. (2022) isolated a leucine-rich lumen binding protein of 24 kDa from *Solanum trilobatum* leaves by ammonium sulfate precipitation and ion exchange chromatography, which was found to have antibacterial activity and edible properties, and can be used for the clinical treatment of *S. aureus and V. cholerae* infections to alleviate the bacterial drug resistance. Ion exchange chromatography can also remove various impurities such as target protein variants, host cell residual proteins, DNA, culture medium components, endotoxin, and viruses (Saraswat et al., 2013; Tripathi, 2016; Kimia et al., 2019; Masuda et al., 2019). In recent years, some improved ion exchange chromatography methods have been proposed. For example, Santry et al. (2020) added NatriFlo^®^ HD-Q membrane and interfering agent to the anion exchange chromatography and developed the interference chromatography technology, which can purify high titer, and clinical grade oncolytic virus by using the difference of molecular bonding interaction, resulting in realizing the large-scale production of oncolytic virus and promoting the application of oncolytic virus in tumor immunotherapy. Jing et al. (2021) adopted a new double column continuous flow chromatography (called the new N-rich mode), that is, strong cation chromatography column (SCX) and weak cation chromatography column (WCX) were used respectively, and the buffer solution system, flow rate and elution gradient were optimized at the same time. After 22 cycles, enrich acidic variants of an IgG1 mAb with a purity of nearly 100% was obtained, significantly improving the purity of the target proteins.

#### 6.1.3 Affinity chromatography

**FIGURE 7:**
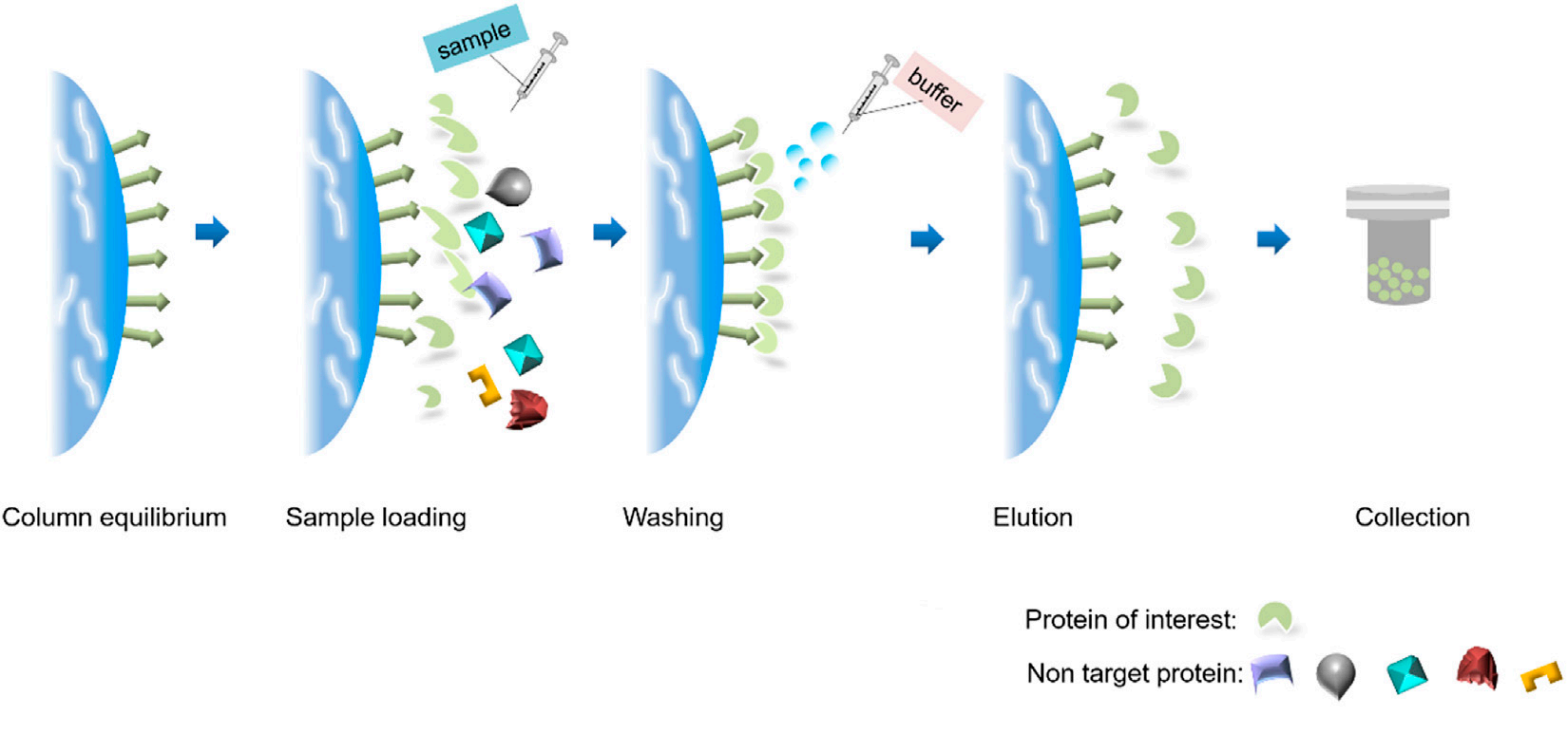
Affintiy chromatography process.

Affinity chromatography (AFC), also known as liquid chromatography, is the most selective technology developed in the 1960s. The principle of affinity chromatography is that one or a group of proteins can selectively and reversibly combine with specific ligands, and the separation of target proteins can be realized according to the different binding abilities of different proteins to specific ligands. Affinity ligands with special structures are usually fixed in the column as stationary phase carriers, forming the basis for affinity column separation or purification of complementary targets (Block et al., 2009). When the protein mixture passes through the chromatographic column, some proteins with affinity are adsorbed on the stationary phase carriers (Arora et al., 2017). Conversely, proteins without affinity will flow out directly **(** Figure 7 **)**. The adsorbed protein can be eluted by selecting an appropriate elution buffer and changing the binding conditions. Affinity chromatography is fast, simple, and highly efficient (Lacki and Riske, 2020), which is often used to isolate compounds with specific tags or to study interactions between biological macromolecules. The interactions between proteins and ligands are based on non-covalent interactions such as electrostatic gravity, molecular hydrophobicity, van der Waals forces and hydrogen bonding forces.

The carrier of affinity chromatography should have the following characteristics (Chen et al., 2003): 1) Insoluble in water, but highly hydrophilic; 2) Inert substance with good specificity; 3) There are enough chemical groups for activation; 4) Stable physical and chemical properties; 5) Good mechanical properties, with a certain particle shape to maintain a certain flow rate; 6) Good permeability, with porous network structure for free passage of macromolecular substances; 7) It can resist the damage to microorganisms and alcohols. The commonly used stationary phase carriers include alumina, polyacrylamide gel, dextran gel, cellulose, agarose, metal chelate and so on.

**FIGURE 8:**
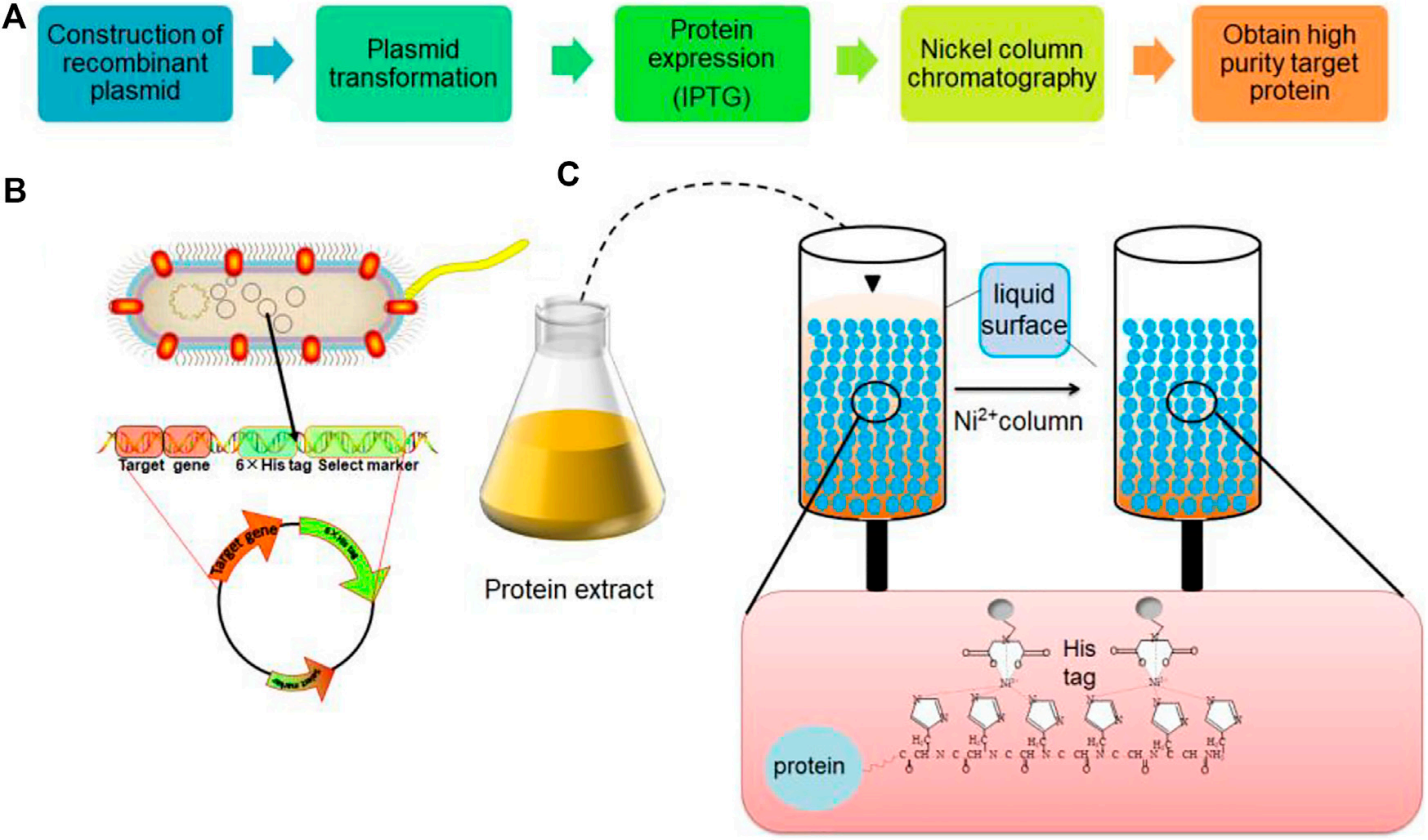
Overall flow chart of nickel column chromatography. **(A)** Overall flow chart. **(B)** Plasmid construction transformation. **(C)** Nickel column chromatography process and his tag structure.

**FIGURE 9:**
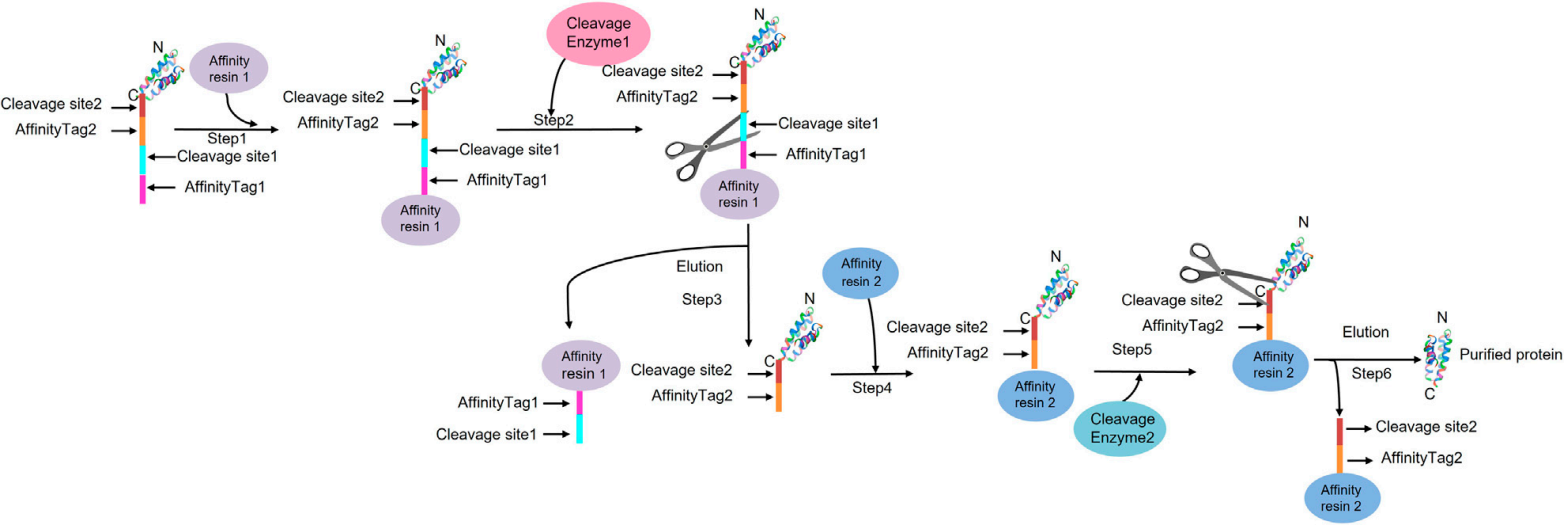
Tandem affinity purification process (Mishra, 2020)

Affinity tags are important recognition structures for affinity chromatography, located inside the N terminus of the target protein, whose essence is a protein or a peptide. Some affinity tags also help the protein fold (Kou et al., 2007), increase solubility (Hammarstrom et al., 2002; Chen et al., 2005; Nallamsetty and Waugh, 2006), and in turn increase the yield of the target proteins (Sun et al., 2005). Currently, multiple types of affinity tags have been developed. The most commonly used His tag (Kinrade et al., 2020) is composed of six or more tandem histidine residues, and it is small, cheap, and has little impact on the structure and function of the target proteins (Figure 8) (Lichty et al., 2005). Abis et al. (2019) purified human soluble cyclooxidative hydrolase by nickel ion chelation chromatography combined with and BTS (Benzylthio-Sepharose) affinity chromatography, and the target proteins showed a high purity, as determined by SDS-PAGE analysis. Ge et al. (2022) purified the recombinant SARS-CoV-2 S1 protein by immobilized metal affinity chromatography and used it as an antigen for immunizing hens. Öztürk and Demir (2021) proposed to use CTS-p (HEMA) -Cu^2+^as an IMAC adsorbent, which can quickly, efficiently and repeatedly detect the amount of residues of melamine in different complex substrates. Wang et al. (2022) used titanium (IV) ion-fixed metal affinity chromatography (Ti^4+^ -IMAC) to jointly extract DNA binding proteins (DBPs) and RNA binding proteins (RBPs), which contributes to the efficient analysis of nucleic acid-binding proteins in cells. Affinity tags can be divided into epitope tags and protein/domain tags, where His, Strep, HA, etc., are often used as epitope tags, GST, MBP, CBP, etc., are often used as protein/domain tags, At the same time, we also screened some new tags found in recent years (Table 6). The tandem use of affinity tags can successfully purify single proteins or protein complexes (Figure 9) (Mishra, 2020). For example, connecting His_10_ at the C end and twin Strep tag at the N end can purify the complete membrane protein receptor CB2 from *E. coli* (Yeliseev et al., 2017). When the affinity tag interferes with the structure or function of the target proteins, the tag is often removed by enterokinase, factor Xa, thrombin, tobacco etch virus (TEV) or human rhinovirus 3C protease or by introducing an inclusion peptide (Zhao et al., 2013). It was found that the natural protein A in the cell wall of *S. aureus* has a strong specific affinity with the Fc fragment of IgG, and the protein A as an affinity ligand can effectively reduce process impurities, and has become the gold standard for monoclonal antibody purification (Das et al., 2020). Protein A was genetically engineered to produce the alkali resistant recombinant protein A product MabSelect SuRe, and further purified to obtain a bispecific antibody m3s193 BsAb with a purity of more than 95%, which can be used for the treatment of gastric cancer (Chen et al., 2021). Therefore, using MabSelect SuRe or developing more potential recombinant protein A products and producing more antibodies against different cancers will effectively improve cancer cure rates and help the biomedical and health industries.

**TABLE 6:** Characteristics and applications of the common affinity tags.

Tags	Sequence/Size	Features	Application
Epitope Tags			
His	6–10 His/0.84 kDa (Mishra, 2020)	It has a small molecular weight, and no impact on the function and downstream application of the target proteins. It has low immunogenicity so that purified proteins were directly immunized to animals and antibodies were prepared (Freitas et al., 2022)	Bromberg et al. (2022) found that His tag can be used as a decoy modulating preferred orientation in cryo-EM, providing a new scheme for improving cryo-EM sample processing
			Williams et al. (2021) used the optimized His tag the integrate with recombinant proteins, which is not only convenient for affinity chromatography, but also provides an effective binding site for the radiolabeling of the recombinant protein. Therefore, the optimized His tag are used in the process of cancer molecular imaging and radionuclide therapy with more economic advantages
FLAG	DYKDDDDK/1.01 kDa (Mishra, 2020)	It has a small molecular weight, high hydrophilicity (Schmidt et al., 2012), The tag has high sensitivity and are easy to remove after use	Han et al. (2017) purified functional reprogramming factors from HEK293 cells using FLAG tag. Imagawa et al. (2021) purified virus-like particles with FLAG-tagged envelope protein through one-step affinity purification with FLAG tag, which could be used as a tetravalent dengue vaccine candidate
HA	YPYDVPDYA/1.1 kDa	The tag is derived from the influenza virus hemagglutinin (Zhou et al., 2012). The tag has little influence on the spatial structure of the target protein, and it is easy to be constructed into tag proteins fused to the N or C terminus	Vyas et al. (2020) CRISPR-Cas9 gene editing technology was used to connect HA tag as locators to the C-terminus of mouse nicotinic acetylcholine proteins (nAChRs) α9 or α10. The expression of α9 and α10 proteins can be detected
Strep	Including Strep-tag II (WSHPQFEK) (Tremante et al., 2021), Twin-Strep-tag (WSHPQFEK- GGGSGGGSG-SA-WSHPQFEK) (Schmidt et al., 2013)	Strep-tag II is small, which does not affect the folding ability of protein, and has strong specific binding ability with affinity resin, which effectively avoids non-specific binding. Therefore, the purity of the target proteins obtained is high. The target proteins can be purified under mild conditions to maintain its activity (Johar et al., 2017)	Kawaguchi et al. (2019) purified highly purified recombinant human pancreatic lipase (recHPL) in the *E. coli* expression system using Strep-tag II. Twin Strep tag retains many advantages of Strep tag II, but for Strep tag II, it further improves the link strength between the target protein and Strep Tactin, and improves the purification efficiency (Schmidt et al., 2013). Xiong et al. (2019) used the high affinity between Twin-Strep-tag and streptavidin variants to establish a rapid separation method for lysosomes, mitochondria and peroxisome organelles, and studied the functions of these organelles after the purification. Yang et al. (2022) used Twin-Strep-tag to construct the TST-EGFP-GPI_BY55_ affinity cell sorting system, which was able to efficiently separate gene transfer positive cells in a simple, convenient and rapid manner to facilitate gene research
V5	GKPIPNPLLGLDST (Mishra, 2020)	The tag is derived from amino acids at positions 95–108 of the α subunit of RNA polymerase of simian parainfluenza virus type 5. (Huang et al., 2011)	Traore et al. (2021) used V5 tag and His_6_ tag to isolate HupZ-V5-His_6_ protein from group A *streptococcus*, and the analysis found that the binding of heme to HupZ was an O_2_-dependent process
Avi	GLNDIFEAQKIEW (Verma et al., 2018)	Almost all proteins can be easily and efficiently biotinylated at a unique Avi Tag site, either *in vitro* or *in vivo*. Biotinylation is achieved by the reaction of the enzyme and the substrate, with mild reaction conditions and highly marked specificity. The effect on the protein space structure is very small	Kim et al. (2021) fused the modified ubiquitin into Avi-tag, and using *in vitro* reconstituted E1-E2-E3 reactions that recapitulate the endogenous mono-ubiquitination reactions, we were able to isolate the monoubiquitin protein through biotin affinity purification and elution by proteolytic cleavage of Avi tag
Myc	EQKLISEEDL/1.2 kDa (Mishra, 2020)	The tag is derived from peptide 410–419 of human c-Myc protein, which can be fused to the N-terminal or C-terminal of the target protein	Myc tag can be used as epitope tag, widely used in the detection of anti-c-Myc hybridoma E10 antibody Myc1-9E10 (Schuchner et al., 2020)
Protein/domain tags			
Glutathione S-transferase (GST)	26 kDa (Williams et al., 2020)	The tag was derived from *Schistosoma japonicum*, which could be expressed in different hosts and was widely applicable. It can be expressed in different hosts, and has a wide range of applications. Different proteases can be easily removed (Kim et al., 2021). It can increase the solubility and improve the expression and stability of foreign proteins. It has good specificity and helps to maintain the antigenicity and the activity of the proteins after eluting under mild and non-denaturing conditions. It has a large molecular weight and may affect protein functions and downstream experiments	Zhou et al. (2020) fused melittin (MET) with the GST tag and expressed melittin using the *E. coli* expression system. After affinity chromatography, digested with prescission protease, gel filtration chromatography, melittin with a purity of >90% was purified, which also showed strong antibacterial activity. Such a scheme could be used for large-scale industrial production of melittin
MBP	40 kDa (Greenfield et al., 2020)	It is one of the most commonly used crystallization chaperones for the target proteins (Fox et al., 2001), and can increase the solubility of the fusion proteins, especially the eukaryotic proteins. MBP tags can be easily detected by immunoassay	Nosaki et al. (2021) have successfully revealed the crystal structure of AtBIL1/BZR1 DBD and target DNA complex by the combination of MBP-mediated crystallization and MD simulation. This is shown to be capable of deciphering the protein-DNA recognition code of interest
			Li et al. (2019) found that MBP tag can assist in the crystallization of the CAMP factor of *Streptococcus agalacia*e, facilitating the subsequent study of the structure of the CAMP factor
			Guo et al. (2018)used MBP tag to prepare high purity and high activity MBP-MLIF in the *E. coli* expression system. On the whole, this method has advantages such as simplicity and application, which promotes the scientific research of stem cells
SUMO	11.2 kDa (Heinrich et al., 2019)	It can increase the expression amount of the fusion proteins (Mohammadi et al., 2022), and has the functions such as anti-protease hydrolysis, promoting the correct folding of the target protein, enhancing the solubility, and shielding the toxic proteins	Kim et al. (2019) used SUMO tag and His tag to construct a new vector pKSEC1 for the production of antimicrobial peptide-Abaecin, providing a new scheme for the treatment of bacterial infection
			Mohammadi et al. (2022) used SUMO tag to express a large number of recombinant human angiotensin-converting enzyme 2 (rhACE2) in *E. coli*, and the solubility of recombinant human angiotensin-converting enzyme 2 (rhACE2) could be improved in combination with freeze-thaw method, providing a reasonable choice for subsequent large-scale purification
Halo	33 kDa (Freidel et al., 2016)	Halo Tag monomeric proteins can be fused to either the N or C terminus of the recombinant proteins and express in prokaryotic and eukaryotic systems. Halo Tag can rapidly bind to ligands with high specificity and affinity to form stable covalent compounds	Deo et al. (2021) used the self-labeling Halo tag protein as a fluorescent indicator sensor scaffold to detect a single action potential in cultured neurons. Liu et al. (2018) connected the Halo tag and tobacco Etching virus (TEV) protease cleavage site at the n-terminal in the *E. coli* expression system. After chromatography and enzyme digestion, high purity Halo-FGF7 and rhFGF7 were respectively determined by *in vitro* and *in vivo* activity, MTT, the protective effects of rhFGF7 and Halo-FGF7 on acute liver injury were further investigated by Western blot. Akin et al. (2021) used Halo tag to mark Nav1.7 channels in nerve cells to study the effect of paclitaxel (PTX) increase axonal localization and vesicular trafficking of Nav1.7
SNAP	19.4 kDa (Freidel et al., 2016)	The tag is derived from human O6-alkylguanine-DNA-alkyltransferase (Padayachee et al., 2019). It can covalently bind with the substrate to tag proteins with biotin or fluorescent groups; highly specialized, stable and suitable for protein detection and purification in various environments	Fu et al. (2021) used SNAP as the tag to construct Snap-tagged EGFR/CMC in combination with HPLC-IT-TOF-MS to screen EGFR-targeted active compounds in traditional Chinese medicine, efficiently isolate and accurately identify them, and screen out important candidates for disease treatment. Moinpour et al. (2022) used the SNAP-tag biocoupling strategy to form stable covalent bonds between benzyl guanine (BG) -modified phospholipids and SNAP-tag fusion proteins, thus realizing programmable control of protein capture. It is also proved that the SNAP-tag biological coupling strategy can directly isolate proteins in the presence of complex biochemical mechanisms, which will facilitate the development of advanced lipid-based artificial organelles
Calmodulin-binding peptide (CBP)	RWKKNFIAVSAANRFKKIS (Mukherjee et al., 2015)	The tag is derived from the 26 amino acid sequences of myosin light streptokinase of skeletal muscle (Wyborski et al., 1999), with a small molecular weight. After purification of the target protein, the tag can not be removed (Zhao et al., 2013), It has the ability to improve the antigen solubility (Mukherjee et al., 2015)	Viala et al. (2017) studied the interaction between proteins by using tandem affinity purification technology consisting of CBP and two immunoglobulin G (IgG) binding domains of *Staphylococcus aureus* protein a (ProtA) isolated by the cleavage site of tobacco corrosion virus (TEV) protease
NusA	54.9 kDa (Dudenhoeffer et al., 2020)	Tag can increase the solubility of the target proteins	Hara et al. (2021) used NusA tag to dissolve and concentrate carotenoids
New type of tag			
XXA	192aa	The synthetic Chlorella sorokiniana antifreeze protein (Accession number: PRW45461) has excellent water solubility and can improve the solubility of the target proteins (Xie et al., 2022)	Xie et al. (2022) used XXA tag as a soluble tag to express Chrono, Notch2NL, nClu, bdNEDP1, NbALFA and other proteins in the prokaryotic expression system
Affinity Bioorthogonal Chemistry (ABC) Tags		The tag is a derivative of 3-methyl-6-(2-pyridinyl) tetrazine	ABC tag has dual functions, which can not only promote protein purification, but also serve as the follow-up arm of rapid and quantitative biological orthogonal labeling (Scinto et al., 2022)

#### 6.1.4 Hydrophobic chromatography

**FIGURE 10:**
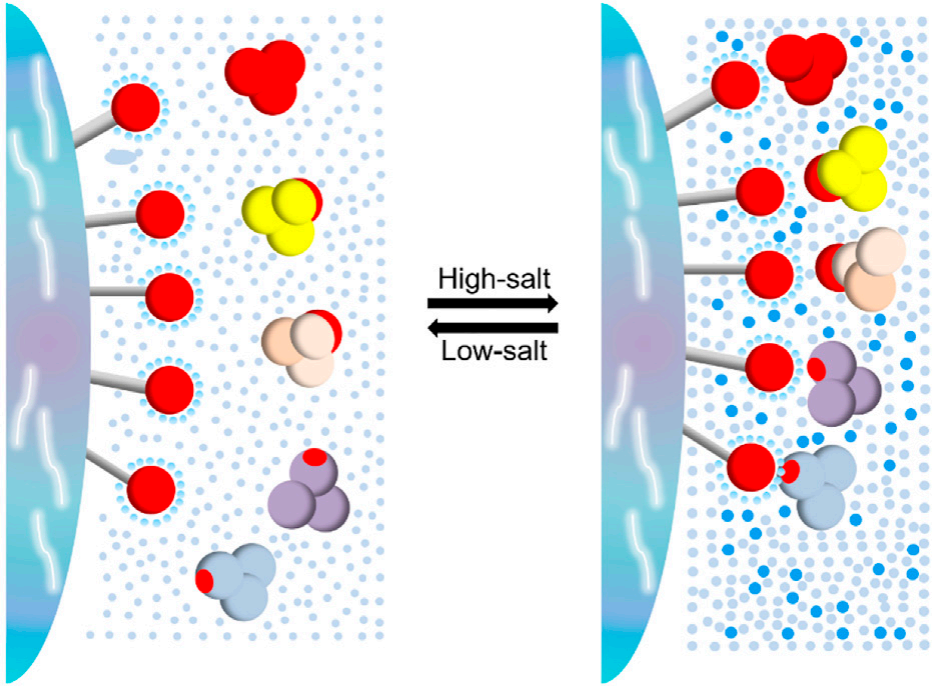
Regulation of salt ion concentration on hydrophobicity balance.

There are some amino acids with hydrophobic side chains on the surface of most proteins, such as phenylalanine, tryptophan, methionine, etc. The number, size and distribution of these amino acids determine the properties of proteins (O'Connor and Cummins, 2017). Hydrophobic chromatography is a purification technology developed based on the hydrophobicity difference of proteins. The balance of hydrophobicity is mainly controlled by salt **(** Figure 10 **)**. Generally, controlling the concentration of salt ions can effectively remove the self-aggregation or self-interaction caused by hydrophobic interaction within the sample. Hydrophobic chromatography can also be used to remove impurities and monitor the purity of the target proteins. Weigel et al. (2019) used hydrophobic chromatography to reversibly combine virus particles to remove residual contaminated DNA and proteins when purifying influenza A and B viruses.

## 7 Challenges and future perspectives

Protein is the material basis of life activities. With the continuous development of molecular biology, structural biology, genomics and bioinformatics, people gradually realize that it is far from enough to clarify the phenomenon and the nature of life activities only by genome sequence analysis. It is also necessary to study life activities from the perspective of proteomics. Only by integrating multi omics can we better understand the phenomena and laws of life, and then reveal the nature of life. Protein isolation and purification is an important technology in the field of biochemistry. At present, it has made unprecedented progress in food, medicine, agriculture, fermentation, textile and other fields. Since proteins are often exist in complex mixtures in tissues or cells, host contamination, sample solubility, structure integrity, purity and biological activity of proteins make purification a delicate and complex task. Therefore, efficient protein purification technology is the basis and key of protein related research.

Protein production has been facing great challenges. Firstly, the establishment of the protein purification scheme requires repeated trials, constant exploration, tedious steps and long cycle, which increases the risk of loss of protein activity. Secondly, the acquisition of high-purity target proteins requires the cooperation of multiple devices, which increases the research cost. Thirdly, other biological macromolecules other than target proteins are often treated as impurities. In fact, the recycling of these substances is of great significance in the field of life and health. Finally, the isolation and purification of membrane proteins has always been a difficult problem in the field, and how to efficiently separate and purify various membrane proteins is still a valuable topic. Therefore, shortening the production process, reducing the production cost, and building a recycling system to recycle valuable substances without destroying the activity and yield of the target proteins are the directions for further development in the future.

At present, the bio health industry has become the focus of global attention, and is bound to be the next explosion point of economic development. With the continuous improvement of people’s living standards, people’s desire for a healthy life has become increasingly urgent. The most promising branches of the life health industry mainly include: second-generation sequencing, *in vitro* diagnosis, immunotherapy, stem cell therapy, biopharmaceutical, etc. The most critical steps in the second-generation sequencing process of library building, capture, and sequencing require the participation of a wide variety of enzymes or proteins. Moreover, the most important factor determining the detection accuracy of various biochemical detection reagents used by major hospitals and third-party detection institutions is the quality of antibodies, antigens or enzymes in these kits.

In recent years, immune and stem cell therapies have been developing rapidly. It is the wide variety of protein-like cytokines that play a key role in the process of cell culture, proliferation and activation, and these factors are key to the success of this therapeutic technique. Most biopharmaceutical molecules are proteins themselves, so the manufacturing cost and the successful development of dosage forms of these drug molecules are usually directly related to the success of this new drug research and development project. In the process of research, development and production of leading biopharmaceutical molecules, it is also inevitable to require the participation of some enzymes and protein molecules. Sometimes the activity or cost of a key enzyme can determine the life and death of this biopharmaceutical research and development project. Insulin, for example, requires two enzymes in its production process. Therefore, successful control of the production of these key raw materials enzymes will better benefit mankind.

In summary, although protein-related products are in the upstream of the life and health industry, it is this upstream that firmly affects the trend and direction of the entire industry. It can be seen that the core competitiveness of the above fields lies in a variety of core protein products upstream. Therefore, how to better accelerate the development of life and health in the post epidemic era is what people expect, and how to better apply the progress made in the protein field to protect people’s life and health still requires our continuous efforts.

## Abbreviations

PEG, Polyethylene glycol; AFC, Affinity chromatography; PEGMA, Polyethylene glycol methacrylate; IPTG, Isopropyl-beta-D-thiogalactopyranoside; BTS, Benzylthio-Sepharose; DTT, DL-Dithiothreitol; DDM, n-dodecyl-β-D-maltopyranoside.
